# P-1959. B.1.617.2 Outbreak Mitigation on an International Steel Cargo Ship Utilizing a Multidisciplinary Approach

**DOI:** 10.1093/ofid/ofae631.2118

**Published:** 2025-01-29

**Authors:** Yasmeen Mann, Mariia Numi, Ryan A Edwards, Joyce Lai, Seema Joshi, Pragnesh Patel, Heather M Blankenship, Marty K Soehnlen, Marcus Zervos, Michael Mossing, Najibah K Rehman

**Affiliations:** Henry Ford Hospital, Detroit, Michigan; Henry Ford Hospital, Detroit, Michigan; Carnival Cruise Line, Sterling Heights, Michigan; Michigan Department of Health and Human Services, Wayne, Michigan; Henry Ford Hospital, Detroit, Michigan; Wayne state university college of medicine, det, Michigan; Michigan State University/Michigan Department of Health and Human Services, East Lansing, Michigan; Michigan Department of Health and Human Services, Wayne, Michigan; Henry Ford Hospital, Detroit, Michigan; Wayne State University, Franklin, Michigan; Henry Ford Health System, Detroit, Michigan

## Abstract

**Background:**

The SARS-CoV-2 B.1.617.2 (Delta) variant emerged in India and became widespread in the United States in 2021. Evidence suggested it was more transmissible than other variants.

**Figure 1:**
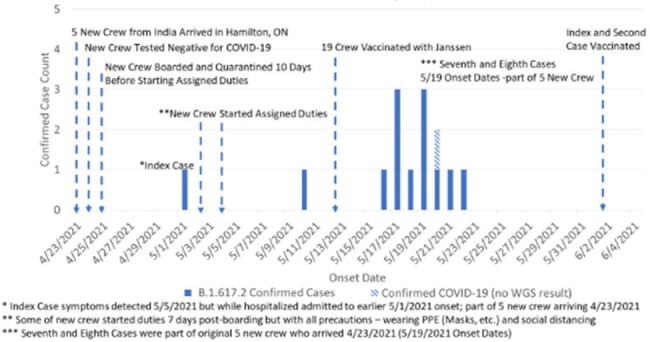
SARS-CoV-2 Cases on the Steel Cargo Ship Timeline

**Methods:**

From 01 May to 22 May 2021, 14 cases of COVID-19 were linked to an international steel cargo ship docked on the Detroit River, after 5 new members joined the crew from India. Cases were reported to the Detroit Health Department (DHD) on 08 May 2021. A strict weekly testing schedule was maintained by DHD. Control measures included a multidisciplinary partnership between DHD, local universities and hospitals. The multidisciplinary team (MDT) considered environmental health, infection control and behavioral health interventions. Measures for control included testing, and quarantine, vaccine, antibody therapy, and access to hospital care.

**Figure:**
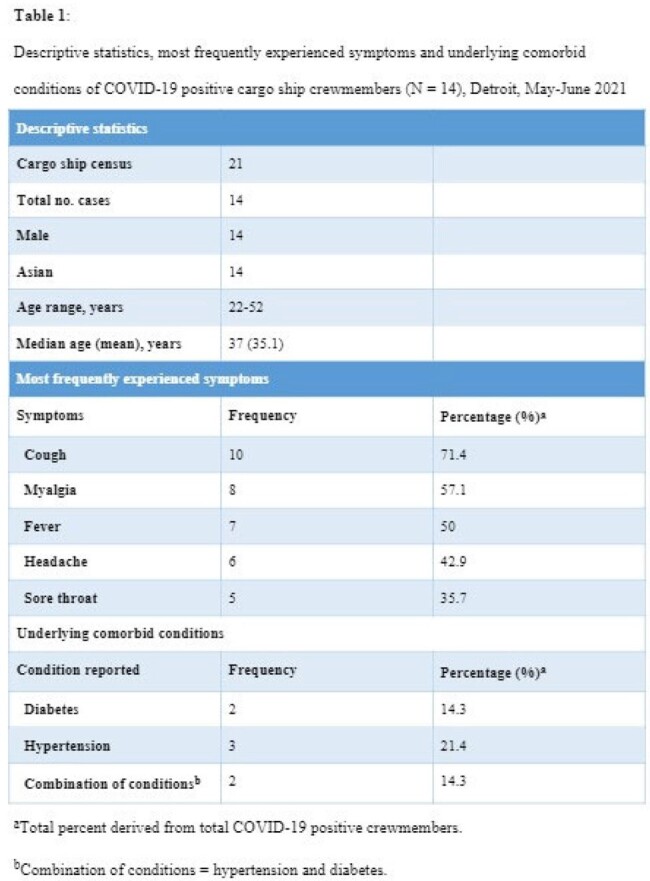

**Results:**

14 out of 21 total crew members were confirmed positive (attack rate 67%). Once no new cases were found, a 14-day working quarantine was applied per the Centers for Disease Control (CDC) guidance. On 13 May 2021, the DHD vaccine outreach team administered Janssen COVID-19 vaccine, without incident, to all medically eligible crew (n = 19). Janssen was selected considering the ship’s upcoming departure from Detroit and the limitation for follow-up doses.

**Conclusion:**

Response measures taken for this ship involved quarantine, isolation, disinfection and ventilation protocols, installation of filter units, behavioral health recommendations for anxiety-reducing strategies, antibody therapy, hospital care, vaccine administration and education. A MDT approach and multicomponent control strategy was critical to mitigate further spread. DHD’s communicable diseases investigations, vaccination, and testing capabilities were essential, including continued guidance from the Michigan Department of Health and Human Services (MDHHS) and CDC. Full transparency from the ship crew prevented potential spread of the variant in Detroit.

On 02 June 2021, the ship was cleared by DHD, MDHHS, and CDC to depart. This incident led to the approval and vaccination of 3 subsequent ships whose crews also originated from the Indian subcontinent. This study provides a model for investigation and control of infection related to international ships.

**Disclosures:**

Seema Joshi, MD, AbbVie: Grant/Research Support Marcus Zervos, MD, Johnson and johnson: Grant/Research Support|Moderna: Grant/Research Support

